# Diabetes and Sepsis: Risk, Recurrence, and Ruination

**DOI:** 10.3389/fendo.2017.00271

**Published:** 2017-10-30

**Authors:** Lynn M. Frydrych, Fatemeh Fattahi, Katherine He, Peter A. Ward, Matthew J. Delano

**Affiliations:** ^1^Department of Surgery, Division of Acute Care Surgery, University of Michigan, Ann Arbor, MI, United States; ^2^Department of Pathology, University of Michigan, Ann Arbor, MI, United States

**Keywords:** diabetes, sepsis, septic shock, infections, complications, resource utilization

## Abstract

Sepsis develops when an infection surpasses local tissue containment. A series of dysregulated physiological responses are generated, leading to organ dysfunction and a 10% mortality risk. When patients with sepsis demonstrate elevated serum lactates and require vasopressor therapy to maintain adequate blood pressure in the absence of hypovolemia, they are in septic shock with an in-hospital mortality rate >40%. With improvements in intensive care treatment strategies, overall sepsis mortality has diminished to ~20% at 30 days; however, mortality continues to steadily climb after recovery from the acute event. Traditionally, it was thought that the complex interplay between inflammatory and anti-inflammatory responses led to sepsis-induced organ dysfunction and mortality. However, a closer examination of those who die long after sepsis subsides reveals that many initial survivors succumb to recurrent, nosocomial, and secondary infections. The comorbidly challenged, physiologically frail diabetic individuals suffer the highest infection rates. Recent reports suggest that even after clinical “recovery” from sepsis, persistent alterations in innate and adaptive immune responses exists resulting in chronic inflammation, immune suppression, and bacterial persistence. As sepsis-associated immune defects are associated with increased mortality long-term, a potential exists for immune modulatory therapy to improve patient outcomes. We propose that diabetes causes a functional immune deficiency that directly reduces immune cell function. As a result, patients display diminished bactericidal clearance, increased infectious complications, and protracted sepsis mortality. Considering the substantial expansion of the elderly and obese population, global adoption of a Western diet and lifestyle, and multidrug resistant bacterial emergence and persistence, diabetic mortality from sepsis is predicted to rise dramatically over the next two decades. A better understanding of the underlying diabetic-induced immune cell defects that persist following sepsis are crucial to identify potential therapeutic targets to bolster innate and adaptive immune function, prevent infectious complications, and provide more durable diabetic survival.

## Introduction

The Third International Consensus Definitions for Sepsis and Septic Shock Report defines sepsis as life-threatening organ dysfunction caused by a dysregulated host response to an infection. This is associated with a >10% in-hospital mortality. Septic shock is defined as sepsis associated with profound circulatory, cellular, and metabolic abnormalities. Patients with septic shock have serum lactate levels >2 mmol/L (>18 mg/dL) and require vasopressors to maintain a mean arterial pressure of 65 mmHg or greater in the absence of hypovolemia. Compared to sepsis alone, it has a much higher in-hospital mortality rate of >40% (1).

Long-term sepsis mortality is abysmal at 60–80%. Despite substantial advances in immune pathophysiology, this number has not considerably improved (2). In intensive care units, sepsis remains the leading cause of death (3). Considering the rapidly expanding elderly population with extensive comorbid burdens, physiological frailty, and immune senescence (4), over the next couple of decades, sepsis mortality is predicted to rise at a frightening rate (5). Just as terrifying are the mounting costs associated with treating septic patients. The United States spends ~$17 billion on sepsis-associated medical care (6).

Despite over 100 therapeutic clinical trials in sepsis, there are no current FDA-approved therapies that improve sepsis survival (7). In contrast, advancements in clinical treatment protocols (8) have resulted in increased in-hospital survival from life-threatening sepsis and organ dysfunction. However, a substantial portion of these in-hospital survivors will then die in the months to years following the acute event. A trimodal pattern of death during and after sepsis has been described. The first peak occurs at several days and is likely secondary to inadequate resuscitation. The second peak occurs at several weeks and is secondary to persistent organ injury and/or failure (9). The late (months to years) deaths comprise the largest mortality group and are speculated to be the consequence of improvements in intensive care medicine that keep elderly and comorbidly challenged patients alive despite persistent immune, physiological, biochemical, and metabolic aberrations (10). In 2008, over 800,000 Medicare patients survived admissions for severe sepsis. This population of survivors is composed of individuals with significant comorbidities that are at risk for hospital readmission (11). Several reports suggest that it is the synergistic effect of patients’ advanced age, comorbidities, and persistent organ injury that create this damaging state of ongoing immune dysfunction, immune suppression, catabolism, and inflammation (12–14), leading to long-term sepsis mortality. Patients with Type II diabetes (T2D) are physiologically frail and comprise the largest population of patients who experience post-sepsis complications and rising mortality.

Type II diabetes is a common and devastating disease frequently encountered by clinicians who care for critically ill patients. With increasing globalization of the western diet and lifestyle, the worldwide incidence and prevalence of T2D is approaching pandemic proportions. In the United States, the prevalence has almost doubled from 11.9 million in 2000 to 21.9 million people in 2014, and the incidence has more than tripled from 1980 to 2014 (15). Globally, T2D is no longer a disease of high-income countries. In 2014, an estimated 422 million adults worldwide had T2D, compared to 108 million in 1980. The largest growth in prevalence can be found in low- and middle-income countries (16). From 1980 until 2014, China, India, and United States had the largest T2D patient populations. However, recently, the global share of people with T2D has dramatically increased in India and China while United States share has decreased. As the growth trends in T2D prevalence continue, the number of adults with T2D will surpass 700 million worldwide in the near future (17).

As medical management strategies improve, patients with T2D live longer with their disease. In addition, the increasingly young age at diagnosis results in prolonged exposure to glucolipotoxicity, low-grade inflammation, and increased oxidative stress, creating a metabolic milieu conductive to cancer growth (18). This represents a major public heath challenge. Delayed diagnosis, inadequate follow-up, and suboptimal care of T2D patients predisposes them to develop acute and chronic complications, leading to further burden on the patient, health-care system, and society as a whole (19). A 2012 global systematic analysis of disease and injury epidemiology identified T2D as a leading cause of years lived with disability (YLD), with a 67.2% increase in YLD from 1990 to 2010 (20). Furthermore, T2D has been shown to be a significant cause of mortality. Stokes and Preston performed a cohort study of National Health Interview Survey and National Health and Nutrition Examination Survey participants between 1997 and 2010 and estimated the proportion of deaths attributable to T2D to be 11.5–11.8% (21). These numbers underestimate the burden of T2D, as an estimated one in four people with T2D are unaware that they have the disease (22). As the sedentary, calorie-rich western lifestyle continues to infiltrate the global landscape, T2D will continue to become a more common comorbidity encountered in the hospital setting.

Patients with T2D have an increased risk of developing infections and sepsis. Although a few rare infections such as *Klebsiella* liver abscesses, malignant otitis externa, and emphysematous cholecystitis are strongly associated with diabetic patients, most infections that occur in diabetics are also common in the general population (23). T2D also worsens infection prognosis, with T2D patients showing increased morbidity and mortality from sepsis (24). The combination of increased incidence, prevalence, and life expectancy of individuals with T2D, combined with an increased risk of infections is resulting in a rapidly expanding patient population consuming more medical resources.

Some investigators have refocused their efforts to work on understanding the underlying innate and adaptive immune system derangements that facilitate the development of infectious complications, impair recovery from sepsis, and increase long-term mortality (25, 26). However, little effort has focused on the interplay between T2D, sepsis, immunity, and their impact on overall survival. In this review, we highlight the immune system’s interdigitating role in the pathogenesis of T2D and sepsis. We focus on the clinical implications and then explore potential therapeutic interventions available to improve long-term survival in patients with T2D. To combat this pandemic, we hypothesize that disease-modifying therapeutics that have the ability to alter the course of disease have to be utilized, instead of focusing on palliative treatments that merely treat the sequelae of disease. Immune-modulatory therapy has been shown to improve patient survival in cancer, autoimmune diseases, and HIV. However, from these successful therapeutic advances, it has been shown that these therapies need to involve multiple agents, given in combination and introduced at the correct time to dampen disease progression, enhance patient immune responses, and affect host–pathogen interactions. We believe single-agent interventions are the reason why the sepsis literature is littered with failed therapeutic interventions. Combine the immune aberrations in T2D with the immune dysregulation found in sepsis and there are multiple targets for modulatory therapy. We propose that combinations of tailored interventions that focus on specific immune system perturbations that exist in sepsis and T2D will result in a high probability of success.

## Immune Dysfunction in T2D and Sepsis

Type II diabetes is a complex clinical syndrome, depicted by persistent hyperglycemia in the setting of decreased insulin secretion and sensitivity, which results in a compilation of aberrant metabolic changes (24). Key metabolic changes include increased formation of advanced glycation end products (AGEs), activation of protein kinase C isoforms, and increased flux through the polyol and hexosamine pathways (27). These changes lead to increase production of superoxide (28), which activates inflammatory pathways, linking T2D to perturbations of the immune system (28). In addition, individuals with T2D have been shown to have abnormal host responses, including disorders of humoral immunity, defects in neutrophil function, and response of T cells (23, 29, 30). A recent study looking at obese individuals with and without T2D showed that individuals with T2D have specific immunological perturbations compared to metabolically healthy obese individuals, supporting the notion that T2D itself contributes to this identified immune dysfunction (31).

There is considerable clinical evidence that T2D worsens prognosis of pathological infections, with increased mortality from infections and sepsis in patients with T2D (24, 30, 32). This raises the pivotal question: why? The hematopoietic compartment constantly replenishes terminally differentiated innate and adaptive cells that are necessary for wound healing, successful tissue regeneration, and immune surveillance against offending pathogens (9). Sepsis impacts the immune system globally by affecting the lifespan, production, and function of innate and adaptive immune cells, leading to homeostatic perturbations in immune cell repletion (33, 34). In patients with T2D, this homeostasis may be altered secondary to over-nutrition and increased adiposity (35). These metabolic-induced immune perturbations clearly play a substantial role in the increased frequency, severity, and duration of infections (24, 28, 36).

**Figure 1:**
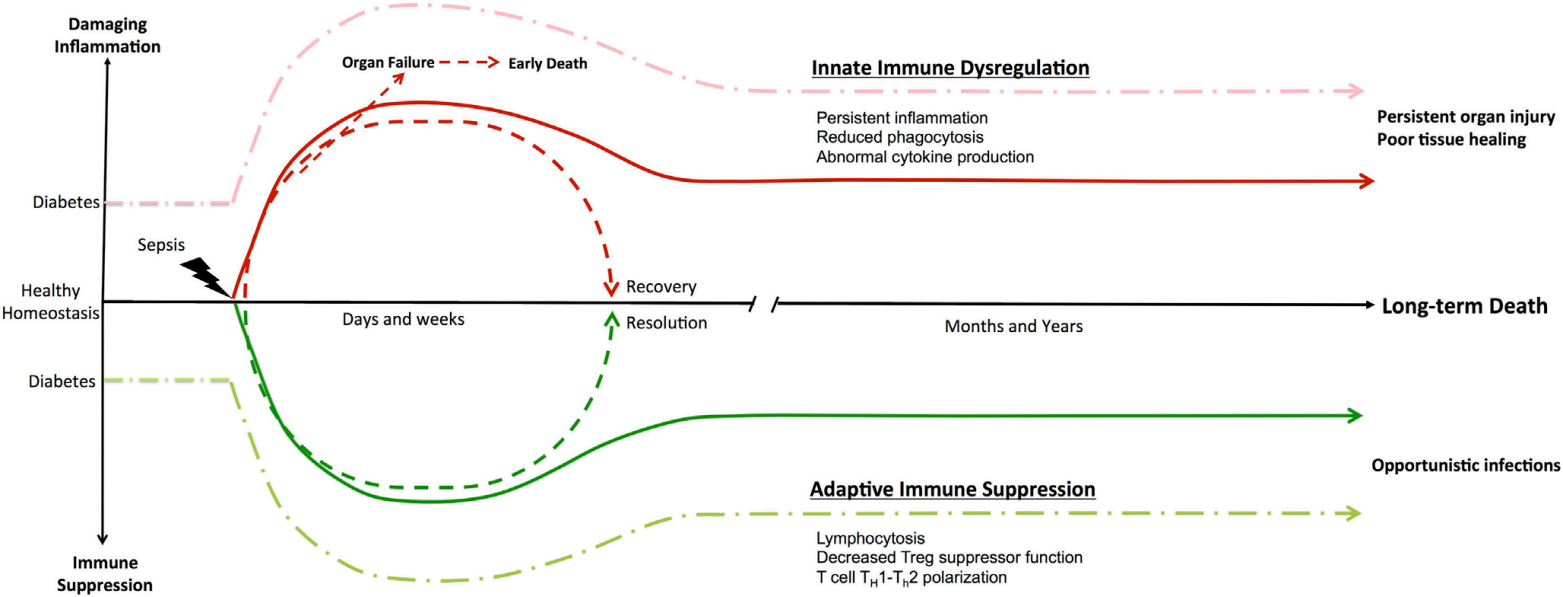
Immune dysregulation in Type II diabetes and sepsis. Diabetes is a functional immune deficiency with chronic inflammation and immune suppression that affects an individuals’ overall immune system homeostasis. The development of patient management protocols in sepsis has decreased early organ failure and sepsis mortality, allowing highly comorbid elderly patients to survive the initial insult. Furthermore, sepsis studies have demonstrated an enduring inflammatory state driven by dysfunctional innate and suppressed adaptive immunity that culminates in persistent organ injury and patient death. Subsequently, the highly comorbid elderly patient population that initially survived now experiences significant morbidity and mortality several months to a year later. Multiple hypotheses for these observations exist, with persistent derangements in the innate and adaptive immune system cellular functions as the main contributors to this long-term mortality.

In sepsis, an ongoing debate persists as to whether inflammatory/anti-inflammatory processes or innate/adaptive immune dysfunction are more detrimental to survival (37). Genomic studies on tissue samples from septic and severely injured trauma patients have provided more information (13). These studies have identified an enduring and simultaneous inflammatory and anti-inflammatory state, which is driven by dysfunctional innate and suppressed adaptive immunity. Together, these culminate in persistent organ injury (38), inflammation, and patient death (39, 40). Figure 1 illustrates how the immune system responds to an acute septic episode. At baseline, patients with T2D have an aberrant immune system. After the initial acute septic episode, T2D patients continue to experience significant morbidity and mortality several months to a year later. We believe that it is the enduring derangements in the innate and adaptive immune system cellular functions that contribute to the long-term morbidity and mortality.

## Metabolic Regulation of Immunity

The immune system protects against foreign microbial invaders, maintains optimal tissue homeostasis, and facilitates wound healing. These processes are dynamic in nature, changing to meet the needs of the organism. Most immune responses are fueled by cellular metabolism that is regulated by extracellular signals, which direct the uptake, storage, and utilization of glucose, amino acids, and fatty acids (9). When the organism senses an invading pathogen or tissue insult, the innate immune cells secrete cytokines, chemokines, and inflammatory mediators, which influence the expansion of adaptive immune cells (9). Since immune cells do not store nutrients, immune responses are only upregulated and sustained when there is an increased uptake of nutrients from the surrounding microenvironment. Nutrients provide substrates for ATP, RNA, DNA, and protein synthesis, along with the membranes necessary for the immune cell’s proliferation and maturation (41). Over a century ago, it was shown that a successful innate effector response is dependent on glucose metabolism (42), and that mitogen-driven proliferation of adaptive immune cells requires the utilization of extracellular glutamine (43, 44). T2D is a disease characterized by aberrant glucose metabolism. Homeostatic conditions are altered with an environment now characterized by chronic hyperglycemia and an increase in free fatty acids (FFAs) (45). An overall change in glucose metabolism therefore contributes to the immune dysfunction seen in T2D and sepsis.

In homeostatic conditions, immune cells rely on oxidative phosphorylation and β-oxidation as energy sources for ATP production (46). However, after stimulation, leukocytes shift their metabolism toward aerobic glycolysis in a process known as the Warburg effect (47). Subsequently, glycolysis produces cellular energy, followed by lactic acid formation in the cytosol instead of oxidation of pyruvate in mitochondria (48). Upon exposure to lipopolysaccharides (LPS), macrophages demonstrate a shift from oxidative phosphorylation to glycolysis and succinate and induce IL-1β production (49, 50). How T2D affects these processes is unknown, but clearly altering the substrates available for these pathways likely contributes to ongoing immune dysfunction. A better understanding of how hyperglycemic environments affect the metabolic checkpoints that control immune cell function, transition, and maturation is needed. In fact, delineating these pathways may provide targets for modulating systemic inflammation, cellular immunity, and recovery from infectious insults suffered by patients with T2D.

While several investigations have addressed the impact of hyperglycemia on sepsis and trauma outcomes in the critically ill in the ICU (51, 52), there is a paucity of studies that address the complications of T2D during infectious states and sepsis. The studies that do examine the association between T2D and sepsis outcomes are limited in their ability to account for all confounders (53, 54). It has been shown that adequate control of hyperglycemia is associated with improved outcomes and survival in times of critical illness; conversely, too tight of glycemic control has been associated with decreased survival (52). This U-shaped curve between glycemic control and mortality suggest that the ideal glycemic control for T2D patients is at moderately elevated glycemic levels. However, it is unclear that this effect is actually due to moderately elevated glucose levels, instead of confounding variables that lead to both lower glycemic levels and poor outcome (55). Although early glycemic control has been associated with risk reduction in the development of heart disease, hypertriglyceridemia, nephropathy, and cataracts, the biochemical mechanisms responsible for these effects are unknown (56, 57). Therefore, the more important question is: does long-term glycemic control augment immune function, prevent infectious complications, and promote durable survival? Although it makes logical sense that early and improves glycemic control would result in better immune function and reduced infections and sepsis episodes, there are few if any studies investigating this assumption. Moreover, there is a paucity of literature investigating the biochemical and physiological pathways central to immune function that benefit from glycemic control. Much more scientific investigation is necessary to determine the biological effect of glycemic control on immune function to improve long-term T2D survival from sepsis.

## Inflammation

Once the host loses local containment of an infection, the body is systemically exposed to microbes, microbial components, and products of damaged tissue. This induces an inflammatory response and initiates sepsis-like responses through the recognition of pathogens and damaged tissue by way of pattern-recognition receptors (PRRs), which are ubiquitous on immune cell surfaces. PRRs are expressed primarily on immune and phagocytic cells and on many types of somatic tissues. Microbial infections are recognized by pathogen-associated molecular patterns (PAMPs), which are expressed by pathogenic and harmless microbes. PAMPs are recognized by PRRs such as toll-like receptors (TLRs), C-type and mannan-binding lectin receptors, NOD-like receptors, and RIG-I-like receptors (9). Proteins and cellular products released by tissue damage are similarly recognized as damage-associated molecular patterns (DAMPs) (58). During sepsis, systemic activation of the innate immune system by PAMPs and DAMPs results in severe and persistent inflammatory responses characterized by an excessive release of inflammatory cytokines such as IL-1β, TNF, and IL-17, collectively known as the “cytokine storm” (38). This unregulated release of inflammatory cytokines occurs over a relatively short period of time (hours or days). Furthermore, instead of stimulating what should be a normal physiological response to an infection, intense complement activation and innate immune cell stimulation enhance an excessive inflammatory response resulting in tissue damage, compromised cellular responses, and molecular dysregulation. The resulting damage incites organ dysfunction and even multiorgan failure (38).

Type II diabetes is an inflammatory disease within itself. In T2D, FFAs bind to TLR2, a receptor for pathogen lipoproteins, and TLR4, a LPS receptor, to activate the innate immune system (59, 60). In addition, there is indirect activation through TLR signaling (61). This elicits the inflammatory pathways activated in sepsis. In addition, AGEs are DAMPs that activate pro-inflammatory pathways.

Several studies also show that the inflammatory response is altered in patients with T2D. For example, mononuclear cells and monocytes have been found to secrete less IL-1 and IL-6 in response to stimulation by LPS, all of which appears to be secondary to an intrinsic defect in cells (29, 62). Although some patients recover from the inflammatory state during an acute septic episode, for unknown reasons elderly patients with significant comorbidities fail to resolve this initial condition. They instead progress to a state of persistent inflammation, immune cell dysfunction, and catabolic metabolism, all of which degrade the immune system’s ability to clear infections and heal injured tissues (63). In individuals with T2D, the chronically inflamed environment may play a role. Adipose tissue serves as a site of inflammation (28), with an increase in adiposity being associated with upregulation of genes encoding pro-inflammatory molecules resulting in the aggregation and accumulation of immune cells (64). Macrophages then create a pro-inflammatory loop by forming crown-like structures, which promote differentiation to pro-inflammatory M1 macrophages (28) and the associated pro-inflammatory cytokines. Similar to the environment seen in adipocytes, pro-inflammatory conditions have also been seen in the pancreas. In the pancreas, there is β-cell apoptosis from glucose-induced IL-1β (65), and β-cell dysfunction by lipoapoptosis from FFAs acting as effector molecules (28). This stress-induced β-cell death results in the release of autoantigens and alarmins, which are endogenous molecules released by necrotic cells resulting in stimulation of the immune system through self-antigen presentation (28). This leads to an enhanced adaptive immune response (66).

Given the growing knowledge in the field of metabolic-induced immune dysfunction in T2D, possible interventions that curb inflammation may offer therapeutic benefits in T2D. In sepsis, recent investigations have suggested that therapeutic interventions that curb hyperinflammation, shift catabolism toward anabolism, and bolster immune function may be beneficial in combination, once the initial episode of sepsis has subsided (25, 67, 68). Although in other disease states, such as severe burns, advanced cancers, and autoimmune diseases, combination therapies that reduce inflammation, optimize metabolism, and decrease infections are common-place, in sepsis there currently is no clear plan for the routine use of these or similar strategies (9). Combinations of immune modulators that target affected pathways in T2D and sepsis have the potential to offer clinically significant improvements in overall survival.

## Molecular Alterations in T2D and Sepsis

The pathogenesis of T2D can be described as insulin resistance associated with inactivity, obesity, and aging (69, 70). Initially, the pancreatic islet cells respond to this decrease in insulin-stimulated glucose uptake by increasing cell mass and secretory activity. When functional expansion of the islet β cells fails to compensate for the insulin resistance, insulin deficiency, and subsequent T2D develop. The hypothesized mechanisms behind insulin resistance and islet β-cells dysfunction focus on molecular changes that influence the pathogenesis of T2D. Specifically, most research centers on lipotoxicity, glucotoxicity, oxidative stress, endoplasmic reticulum stress, amyloid deposition in the pancreas, and ectopic lipid deposition in the muscle, liver, and pancreas (70). The contribution of each of these mechanisms remains unclear, but, interestingly, all of these cellular stresses can be caused by over-nutrition (71) and are induced or exacerbated by an inflammatory response (72).

Obesity-induced inflammation is chronic and indolent, differing from the more acute type of inflammation commonly associated with infections (70). Current observations in sepsis show that sepsis-induced organ dysfunction occurs primarily though cellular and molecular dysregulation of signaling pathways, as opposed to gross tissue damage. This may result in multiple organ failure even in the context of preserved cell morphology and in the absence of significant cell injury. Therefore, immune dysfunction in sepsis is associated with molecular alterations that alter cellular phenotype and function. How the molecular changes in T2D and sepsis interact and influence each other resulting in worse clinical outcomes is unclear. Below we outline several important pathways of cellular dysfunction that impact immune function in diabetics and sepsis, illuminating gaps in knowledge, which could influence why patients with T2D have infections that are difficult to treat and are associated with significant morbidity and mortality (70).

### Complement Activation

Obesity and elevated insulin levels have been associated with elevations in plasma C3 (73), C5, and C8 (74). These increased levels are likely a result of glycated immunoglobulins activating complement (75). Elevated glucose may then attack the thioester bond of C3, making it functionally deficient and leading to a decreased ability to opsonize bacteria (76). In sepsis models, a robust and consumptive depletion of complement occurs, resulting in a sharp drop in the hemolytic activity of plasma complement and its activation products (77). There is also evidence that sepsis in humans causes shedding of the C5a receptor into plasma, likely due to release of microparticles from neutrophils (78). In addition to complement activation in sepsis, there is well-established evidence that activation of the complement system leads to activation of the clotting and fibrinolytic systems (79), resulting in activation of several clotting factors, including thrombin, which have C3 and C5 convertase activities. These ultimately generate C5a and the terminal membrane attack complex (MAC) (80). The progress in understanding how complement activation increases systemic inflammation, organ failure, and mortality have resulted in the development and randomized phase 2 trial of a C5a inhibitor, CaCP29 (EudraCT Number: 2013-001037-40). This C5a inhibitor has shown great promise despite a historically large field of other failed antibody inhibitors (81).

The fact that glycated immunoglobulins affect complement could obviously play a role in T2D patients having an increased risk of infections. However, it is still unclear why these patients have worse outcomes during septic episodes. One hypothesis is that obese T2D patients have baseline elevations of C5, which then becomes activated by enzymatic cleavage during a septic episode, leading to more MAC generation. To date, there have been no published studies looking at C5a inhibitors in T2D patients with sepsis.

### Mitochondrial Dysfunction and Redox Imbalance

Mitochondria are essential for maintaining an adequate supply of ATP for cellular processes. Mitochondria have a significant role in glucose-stimulated insulin secretion from pancreatic β cells (82), with decreases in mitochondrial oxidative activity and ATP synthesis leading to insulin resistance (83, 84). Mitochondrial dysfunction, or direct damage of mitochondria, can trigger cell death pathways through release of mitochondrial cytochrome *c* (9, 85) as well as directly affect the generation of ATP. Not only will the drop in ATP negatively affect intracellular processes and cellular function, such as insulin secretion, but severe lack of ATP can also trigger cellular anergy. In this state, the cell does not necessarily die, but instead acquires a hibernation-like state resulting in tissue dysfunction and organ failure (86).

In addition, hyperglycemia itself has been shown to induce ROS. Obese and insulin-resistant T2D individuals have a hyperglycemic intercellular environment with elevated concentrations of FFAs (87). Hyperglycemia itself has been shown to induce ROS (88, 89) through enzymatic cascades in mitochondria, including activation of NADPH oxidase, uncoupling of NO synthesis, and stimulation of xanthine oxidase (90). Glycated proteins have also been shown to promote ROS formation (91). ROS may then lead to the formation of NLRP3 inflammasomes and caspase 1, which activates the IL-1, pro-inflammatory system (92, 93).

In sepsis, there is generation of excessive amounts of ROS and RNS, which can directly inhibit respiration and damage the respiratory chain components in mitochondria (94–96), leading to mitochondrial dysfunction (9). In addition to this pathway, sepsis-impaired tissue perfusion (due to fluid loss, both intrinsic and extrinsic, as well as reduced vascular tone) leads to tissue hypoxia. Loss of tissue oxygenation significantly impairs oxidative phosphorylation and may trigger cell death pathways (97). In T2D, microvascular dysfunction can lead to local tissue hypoxia. The degree to which local tissue hypoxia propagates cell death and enables ongoing infections in T2D has not been defined.

In both T2D and sepsis, mitochondrial dysfunction and redox imbalance plays an integral role in progression of disease. In human models, cellular ATP levels are correlated with sepsis survival (96, 98). In T2D, changes in cellular ATP levels lead to insulin resistance. In a T2D patient with sepsis, it is unclear if these altered pathways are synergist, antagonistic, or some combination of both. Either way, given the oxidative stress, it seems clear that antioxidant therapies may have a therapeutic role.

### Calcium (Ca^2+^) Homeostasis

Calcium homeostasis in T2D is ubiquitously impaired across tissues, including but not limited to adipocytes, platelets, pancreatic β cells, kidney, and liver (99). The most consistent finding is an increase in intracellular Ca^2+^ levels, leading to tissue-specific dysregulation (99), such as glucose resistance. Glucose homeostasis is determined by the rate of glycolysis, gluconeogenesis, glycogen synthesis, and glycogenolysis, all which are calcium-regulated pathways (100, 101). When intracellular Ca^2+^ increases, glycogen synthase is inhibited causing glucose resistance (102). Clinical trial NCT00436475 examined how Ca^2+^ supplementation impacted pancreatic β cell function, but did not show any significant differences (103, 104).

Hypocalcemia in sepsis, hypothesized to be secondary to defective intracellular calcium homeostasis, is common and correlates with disease-specific scores during critical illness (105). Although systemic Ca^2+^ levels are reduced during sepsis, there are increased cytosolic Ca^2+^ levels, similar to those observed in T2D. These heightened intracellular Ca^2+^ levels lead to elevated inflammatory responses, cellular dysfunction, and can even be cytotoxic (9). In addition, accumulation of Ca^2+^ in organs during sepsis is associated with significant organ dysfunction (106).

### Poly(ADP-Ribose) Polymerase 1 (PARP1) and PARP2 Activation

Poly(ADP-ribose) polymerase 1 and PARP2 are enzymes that catalyze poly(ADP-ribosyl)ation of proteins, after being stimulated by DNA strand breaks. PARP activity is therefore viewed as a sensor of DNA damage. PARP1 activation and initiation of the inflammatory response occur simultaneously (107). PARP1 activity upregulates pro-inflammatory gene expression (108), which is attributed to PARP1-induced alterations in chromatin structure and in transcriptional regulation (107, 109). Because PARP1 also directly contributes to cell death in affected tissues (107) it is hypothesized that PARP1 has a role in sepsis-associated immune cell death. Further data to elucidate the role of PARP enzymes suggests they play a role in metabolic regulation by affecting mitochondrial function and oxidative metabolism (9). PARP activation impacts cellular functions by diverse mechanisms. In general, PARP inhibition enhances oxidative metabolism and mitochondrial content. This suggests that reducing PARP activity may prevent metabolic-related diseases such as T2D, which are characterized by impaired mitochondrial function (110).

Inhibitors of PARP1 have been assessed in clinical trials as potential cancer therapeutics, but trials in sepsis and T2D have not been initiated. It is not clear whether inhibition of PARP1 in humans would be beneficial in the case of T2D or sepsis. In addition, the practicality of long-term inhibition without negative effects on genomic stability is unknown (110).

## Cellular Defects

Below we will summarize the alterations seen in the majority of innate and adaptive immune cells in T2D. Furthermore, we highlight how these cells types are affected by sepsis and try to illustrate how T2D and sepsis together may interact to exacerbate long-term mortality.

### Innate Immunity

#### Endothelium

The endothelium, a single cell semi-permeable barrier, is composed of endothelial cells (ECs), which line all of the vasculature and lymphatic systems in the body. They also play a role in many innate and adaptive immune responses (9). They are one of the first cells to identify invading microbes in the bloodstream *via* endogenous metabolite-related danger signals (111). ECs express TLR-2 and TLR-4, which enable them to be activated by LPS. Activation subsequently leads to the production of pro-inflammatory cytokines and chemokines. These boost the immune response through recruitment of further immune cells (112). Therefore, ECs function as innate force multipliers, cell mobilizers, and immune regulators by modulating cellular function (113). In addition, ECs also express both MHC I and II molecules, which allow them to serve as antigen presenting cells for T cells by presenting endothelial antigens (112).

Endothelial cells are very sensitive to blood glucose alterations, with hyperglycemia-induced ROS leading to EC damage (114). In T2D, increased concentrations of glucose and FFAs also activate ECs, leading to a pro-inflammatory and pro-thrombotic endothelial phenotype (115). There is increase production of plasminogen activator inhibitor-1, thromboxane, tissue factor, and von Willebrand’s factor (vWF), which promotes platelet aggregation and adhesion to the subendothelial layer and the formation of pathological thrombi (116). In sepsis, EC dysfunction is present and manifests as several pathological processes including capillary leak, altered vasomotor tone, and microvascular thrombosis (117). An increased release of pathological quantities of vWF once again promotes platelet aggregation and adhesion to the subendothelial layer and the formation of pathological thrombi. These findings show that ECs are key regulators of the physiological and immune dysfunction seen in both T2D and sepsis. It would make sense that worsened EC dysfunction would be present in a septic T2D patient compared to a septic non-T2D patient given the pathways involved, but how these pathways interconnect is not understood. However, it is clear that EC modulation could be beneficial to improve survival outcomes in septic T2D patient cohorts.

#### Neutrophils

Neutrophils are the most prevalent and integral cell type of innate function and are critical for containment and eradication of microbes (9). Neutrophil dysfunction has been linked to hospital-acquired infections (118). Neutrophils are the majority cell in bone marrow and are the very first responders to microbial infections sites (119). One important aspect is their capacity to produce pro- and anti-inflammatory cytokines and growth factors, which regulate the inflammatory response (120).

In T2D, neutrophils show defects in almost all functions, including migration to inflammatory sites, phagocytosis, release of lytic proteases, production of ROS, and apoptosis (121). In addition, a study evaluating the release of TNF, IL-1β, and IL-8 from neutrophils in individuals with T2D showed increased amounts of TNF, IL-1β, and IL-8 in both the basal state and after stimulation by LPS. This excessive release may lead to tissue injury and cell death (121), increased susceptibility to invasive microorganisms (122), and impairment of normal wound healing (123).

In addition to microbial eradication by phagocytosis, oxidative burst, and degranulation, it has been shown that neutrophils can eliminate a wide range of microbes by forming neutrophil extracellular traps (NETs) (124). If a system is primed to produce NETs, a process termed NETosis, tissue damage can occur (125). NETosis requires a microenvironment with increased levels of TNF (126), upregulated PAD4 (127), elevated intracellular calcium levels, and fasting serum glucose (128), which are all seen in T2D.

In sepsis, there is delayed neutrophil apoptosis (129), leading to ongoing neutrophil dysfunction. This delayed apoptosis is further complicated by the release of immature band-like neutrophils from the bone marrow that demonstrates clear deficits in oxidative burst (130), cellular migration patterns (131, 132), complement activation ability, and microbial eradication (133). These defective neutrophils play a signification role in the persistent inflammation and immune dysfunction seen in sepsis. These findings combined with TLR signaling deficits, chemokine-induced chemotaxis reductions, altered apoptosis signaling pathways, and neutrophil immune senescence, result in a sundry of functional deficits that endure long after sepsis symptoms have subsided (9). In addition, septic patients have been shown to have elevated NET concentrations compared to healthy controls, and that these increased NET levels were associated with sepsis severity and organ dysfunction (84).

Neutrophils clearly have a role in the immune dysfunction seen in both T2D and sepsis. The increased tendency to form NETs contributes to the pathogenesis of both diseases; however, how or if this contributes to the worsen outcomes in patients with sepsis and T2D is unclear.

#### Monocytes and Macrophages

Macrophages have important roles in immune response and homeostasis. They play a significant role in phagocytosis, effectively killing microbes, and in clearing apoptotic and necrotic cells. In addition, they secrete pro- and anti-inflammatory cytokines and express MHC-II molecules, allowing them to activate CD4^+^ T-cells and promote differentiation into T helper subsets (9, 134). Just as important, they play a role in the regulation of glucose and lipid metabolism, and in the inflammation of adipose tissue (135). Macrophages have the ability to display remarkable phenotypic heterogeneity depending on the biological situation (136), leading to the establishment of M1 pro-inflammatory (CD11C^+^) and M2 anti-inflammatory macrophages. First discovered in adipose tissue (64, 137), it was shown that accumulation of macrophages leads to elevated inflammatory cytokines. In addition, the accumulation of these inflammatory cytokines is associated with insulin resistance. The mechanism behind the accumulation of these pro-inflammatory M1 macrophages is thought to occur through two main processes. First, the adipocytes and resident macrophages secrete increased levels of chemokines, LTB3, MIP, MIF, and MCP-3 to promote recruitment of blood monocytes (138). Once the monocytes arrive to the area, the inflammatory signals within the adipose tissue push the monocytes to differentiate into the pro-inflammatory M1 phenotype.

In sepsis, blood monocytes have endotoxin tolerance, with the reduced ability to release pro-inflammatory cytokines after an LPS challenge (9). This has been suggested to facilitate poor short- and long-term sepsis outcomes (139, 140). Although a sundry of complex mononuclear cell signaling pathways are altered and contribute to the establishment of endotoxin tolerance, the major implication on monocytes, and to a lesser extent macrophages, is reduced antigen presentation related to diminished HLA-DR cell surface expression (141). In addition, the reduced monocyte capacity to secrete pro-inflammatory cytokines suggest that intracellular signaling has shifted toward the production of anti-inflammatory mediators, which are associated with hospital-acquired, ongoing, and secondary infections that ultimately increase sepsis-associated mortality. Although the mechanisms accounting for monocyte LPS tolerance are not clear, sepsis-induced monocyte epigenetic reprogramming may play a pivotal role in the establishment of LPS tolerance, myeloid anergy, and the overall immune suppressive monocyte phenotype (142). Analysis of human monocyte mRNA clearly shows increased levels of inhibitory cytokine genes and reduced levels of pro-inflammatory chemokine genes (143).

These findings make us question what happens to monocytes and macrophages in T2D individuals with sepsis. At baseline, obese T2D individuals have a shift toward pro-inflammatory macrophages; however, the fate of these recruited macrophages and their contributions to infection eradication remain less studied. Unlike in a resolving acute infection where homeostasis is restored, adipose tissue inflammation fails to resolve naturally (144). When a T2D individual is exposed to an acute infection, it is unclear how monocyte and macrophage populations change and if these changes are affected by the baseline obesity and chronic inflammation.

#### Natural Killer (NK) Cells

Natural killer cells act as immune complex regulators. NK cells have the ability to destroy target cells spontaneously, without prior exposure, and without MHC restrictions (145). In sepsis, NK cell cytotoxic function is greatly decreased (146) and specific subsets of NK cells are significantly altered. These changes have been associated with increased lethality (147). Recent studies show that individuals with T2D have abnormal NK cell phenotypes, with a significant decrease in NKp46, a NK receptor that recognizes influenza hemagglutinins, and tumor ligand NKG2D, an activating receptor on NK and CK8^+^ lymphocytes. They also have functional defects with reduced degranulation (148). In T2D patients, it is unknown what happens when these altered NK phenotypes are further affected during a septic episode.

#### Dendritic Cells (DCs)

Dendritic cells are characterized as conventional DCs (cDCs) or plasmacytoid DCs (pDCs). cDCs secrete IL-12 and are comparable to monocytes. pDCs secrete IFNα and are comparable to plasma cells. cDCs and pDCs have enhanced apoptosis in patients with sepsis, as well as in patients who developed nosocomial infections (9). In T2D, elevated glucose induces a pro-inflammatory cytokine profile in DCs leading to their maturation (149). It addition, hyperinsulinemia promotes DC activation and upregulation of scavenger receptors including SR-A and CD36, a receptor found on many cells including ECs, cardiomyocytes, platelets, monocytes, and macrophages, all which are involved in the macrovascular complications of T2D (150). AGEs, through binding with SR-A, can also induce maturation of DCs (151).

In sepsis, just like monocytes, DCs have decreased HLA-DR expression and secrete increased amounts of IL-10, which is anti-inflammatory. In addition, when DCs are cocultured with T effector cells, T cell anergy in induced and regulatory T cell (Treg) proliferation enhanced, both which correlate with sepsis-induced immune dysfunction (152). A couple of recent investigations have also demonstrated that inhibition of sepsis-induced DC apoptosis or amplification of DC function improves sepsis long-term survival (153, 154). These observations reveal that adaptations in DCs contribute to the pathogenesis of T2D and sepsis and that targeted manipulation of DCs may provide a therapeutic strategy.

#### Myeloid-Derived Suppressor Cells (MDSCs) and Myelopoiesis

Myeloid-derived suppressor cells are a heterogeneous population of undeveloped myeloid cells. They expand during trauma and sepsis, impede immune responses, and signal through TLR-mediated pathways (155, 156). MDSCs inhibit CD8^+^ T cell function; however, their impact during sepsis is uncertain. Current literature implies a beneficial role, by focusing on their ability to restore innate immune cell function and surveillance through “emergency” granulopoiesis (132). Prior to MDSC increase, there is a brief period of host vulnerability to secondary microbial infections. This brief period is associated with overall mortality secondary to reduced numbers of bone marrow cells and a reduction in neutrophil and monocyte numbers and function (130). It has also been demonstrated that robust MDSC expansion, *via* augmented granulopoiesis, imparts lasting immunity to secondary and nosocomial infections during sepsis (157). Given these findings, there is mounting interest in exploring myelopoiesis, MDSC expansion, “emergency” granulopoiesis, and hematopoietic stem cell (HSC) production and function (130, 155, 157–159). Due to the importance of efficiently regenerating functioning neutrophils, monocytes, and DCs during sepsis, MDSCs expansion is a necessity to replenish the pool of functional innate immune cells. However, in T2D and obese patients, hematopoiesis and myelopoiesis are significantly altered (9). This observation raises the question as to the combined impact of myelopoietic derangement promoting ongoing infection, depressed wound healing, and increased mortality following sepsis.

It has been demonstrated that HSCs and myeloid lineage expansion all occur through c-KIT-, type-I IFN- (IFN-I), and CXCL10-dependent signaling that involves IFN-I-secreting B cells (158, 159). Impaired HSC proliferation, development, and function in human bone marrow transplant and diabetic models (160) is clearly associated with increased mortality from chronic, secondary, nosocomial infections (161). Humans with altered granulopoiesis ability undoubtedly experience more frequent, severe, and anomalous infections, demonstrating the essential requirement for effective neutrophil production especially in T2D (23). Recently, patients with sepsis have been shown to have persistently increased MDSCs that are functionally immune suppressive. These are associated with adverse outcomes including increased nosocomial infections, prolonged ICU stays, and poor functional status at discharge (162). On the other hand, overabundant MDSC proliferation may provoke a physiological state of persistent inflammation, such as in adult respiratory distress syndrome, leading to septic patients having poor outcomes (12). Recent work has demonstrated that acute inflammation causes the reduction of peripheral lymphocytes and common lymphoid progenitors in the bone marrow, which has been connected with a profound reduction in the number of osteoblasts (9). The specific contributions of lymphopoiesis, myelopoiesis, and MDSCs to sepsis recovery in T2D populations versus persistent inflammation and catabolism remain poorly understood. However, new insights into these processes and their roles in sepsis resolution and recovery will hopefully present new targets for immune-modulatory therapy to improve sepsis outcomes in T2D cohorts.

### Adaptive Immunity

#### Lymphoid Apoptosis and Immune Suppression

Apoptosis plays a crucial role is tissue homeostasis and the size and duration of immune responses. Once an infection is successfully cleared, activated lymphocytes undergo apoptosis to curtail the immune response. In the periphery, lymphocyte numbers are tightly regulated. Increased lymphocyte apoptosis leads to immunodeficiency, whereas decreased lymphocyte apoptosis leads to cancer and autoimmune diseases (163). Lymphocyte apoptosis is accepted as a critical step in the pathogenesis of sepsis and contributes to septic immunosuppression (164). It has been shown that T2D patients have an overall leukocytosis; however, analyses of these leukocytes show an overall lymphocytosis (163). Given these findings, blockade of lymphocyte apoptosis may have a therapeutic benefit in septic T2D patients.

#### Gamma Delta T Cells (γδ T Cells)

Gamma delta T cells are a diminutive subset of T cells that have a T cell receptor made up of one γ chain and one δ chain. This uniquely distinct group of T cells exists in the skin, lungs, adipose tissue, peripheral blood, and intestinal epithelium. Once activated, γδ T cells release interferon gamma (IFNγ), IL-17, and other inflammatory chemokines (9).

Obese individuals have a decreased amount of γδ T cells, which is inversely proportionate to body mass index. In addition, the remaining γδ T cells have a reduced ability to secrete IFNγ (165). This is significant because despite obesity being a pro-inflammatory condition, they have a decreased ability to mount an inflammatory response. The number of circulating γδ T cells is also significantly diminished when individuals have an episode of sepsis. Reductions in the γδ T cell population have been correlated with high rates of sepsis lethality (166). These findings suggest that γδ T cells represent a possible target for immune enhancement.

#### T Helper Cell (Th Cell) Subpopulations

T helper cells assist other cell types with immunological processes. APCs present peptide antigens to CD4^+^ cells through MHC class II molecules. The CD4^+^ cells are quickly activated, proliferate, and efficiently secrete cytokines, which modulate adaptive and innate immune responses. Upon activation, CD4^+^ cells have the capability to differentiate into specialized T cell subsets, including Th1, Th2, Th3, Th17, Th22, Th9, or T follicular helper. These subsets promote monocyte stimulation, B cell differentiation, and cytotoxic T cell activation through cytokine generation and secretion (9, 167).

It is hypothesized that adipocytes upregulate class II MHC molecules and play a direct immunological role in antigen presentation (168). Several clinical studies have shown that there is a decline in naïve CD4^+^ T cells, as well as an imbalance of CD4^+^ Th cell subsets toward Th17 and Th22 pro-inflammatory subsets in obese individuals with T2D. This leads to a cytokine-induced hyperinflammatory response leading to further innate immune system activation and response (169). This shift to a pro-inflammatory environment is of significant importance in patients with T2D, as it has been shown that Th cells contribute to the complications associated with T2D, such as coronary artery disease (169).

In sepsis, CD4^+^ populations undergo apoptosis (13, 170). Compared to individuals who survive an episode of sepsis, in humans who die from sepsis there is more lymphocyte (specifically CD4^+^ cells) apoptosis. When evaluating the CD4^+^ cells that survive, there is reduced Th1- and Th2-associated cytokine production both during and long after sepsis subsides (171). In addition, Th17 cytokine production is reduced in sepsis and probably negatively impacts long-term mortality (172). These Th populations play a significant role in both T2D and sepsis. The mechanism by how they contribute is still unclear but it may be that Th cells contribute to the development of the macrovascular complications of T2D, which then contributes to long-term mortality in T2D patients.

#### Regulatory T Cells

Regulatory T cells are master regulators of the adaptive immune system. They help maintain self-tolerance and suppress responses of effector T cells subsets (9). An appropriate balance of pro-inflammatory (Th1 and Th17) and anti-inflammatory (Treg) cells are critical to maintain homeostasis. In T2D, there is a loss of homeostasis with a decreased amount of Tregs (173, 174). This imbalance is hypothesized to contribute to the clinical complications of T2D (175). Tregs have also been shown to induce M2 macrophage differentiation. Therefore, it has been speculated that the decrease in Tregs in T2D contributes to the known polarization toward M1 macrophages.

During the period of inflammation, such as sepsis and critical illness, Tregs enhance the deleterious effector T cell suppression, which subsequently prolongs recovery and may dispose to increased complications. There is an increased Treg ratio present early after episodes of sepsis, which is either due to an absolute increase in Treg number or effector Th cell loss from apoptosis. It could be that Tregs are not susceptible to sepsis-induced apoptosis (176). The fact that hospitalized patients who died from sepsis and T2D patients both have alterations in their Treg amounts make Treg function a possible therapeutic intervention.

#### B Cells

B cells are a very diverse immune cell population. Historically, B cell function was thought to only encompass producing antibodies and plasma cells for long-term antibody responses; however, recent data have focused on the role of B cells in chronic inflammatory disease and sepsis (9). In T2D, TLR ligands activate B cell cytokine production, most significantly IL-8. This pro-inflammatory response then augments T2D patient’s B cell inability to upregulate IL-10 production in response to TLR ligands (177). In *ex vivo* studies in both aging and sepsis patients, B cells demonstrated significant reductions in supernatant IgM production, which may explain why older individuals are more vulnerable to Gram-negative bacteria and fungal infection (178). It is unclear what happens to the B cells in elderly patients with T2D during sepsis, but clearly B cell physiology contributes to the worsened morbidity and mortality experienced by this patient cohort.

## Inflammation Resolution

As related to infection, inflammation is generally followed by inflammation resolution. In sepsis, compensatory anti-inflammatory pathways are activated shortly after sepsis initiation (37). The hallmark cytokine in these anti-inflammatory pathways is IL-10. IL-10 suppresses IL-6 and IFNγ, while stimulating the production of soluble TNF receptor and IL-1 receptor antagonist (IL-1RA). At the subcellular level, autophagy eliminates DAMPs and PAMPs by packaging pathogen components, damaged organelles, and cellular proteins into vesicles targeted for lysosomal degradation. This results in reduced inflammation and cellular activation (179). After a severe infection, resolution of inflammation involves an interdigitating, complex, and coordinated array of cellular processes and molecular signals. The offending pathogen needs to be eliminated from the host, while damaged tissues, cells, and leukocytes need to be removed. These processes occur through activation of anti-inflammatory pathways with production of IL-10 and transforming growth factor β.

Sepsis differs from obesity and T2D since the latter has persistent inflammation that does not resolve. The secretion of pro-inflammatory adipokines [IL-6, TNF, and monocyte chemoattractant protein-1 (MCP-1)] is increased while the secretion of anti-inflammatory and insulin-sensitizing adiponectin is reduced (180). The formation of pro- and anti-inflammatory lipid mediators is also deregulated in obesity (181). In addition, deficiencies in IL-10 expression or IL-10 receptor signaling results in inflammatory diseases (182, 183). A recent study showed that T2D patients have decreased IL-10 function, through downstream signaling in the IL-10 pathway (184). Moreover, expression of IL-1RA is decreased in β cells from T2D patients, with an IL-1RA being a current FDA-approved therapeutic (185).

## Immune Suppression

**Figure 2:**
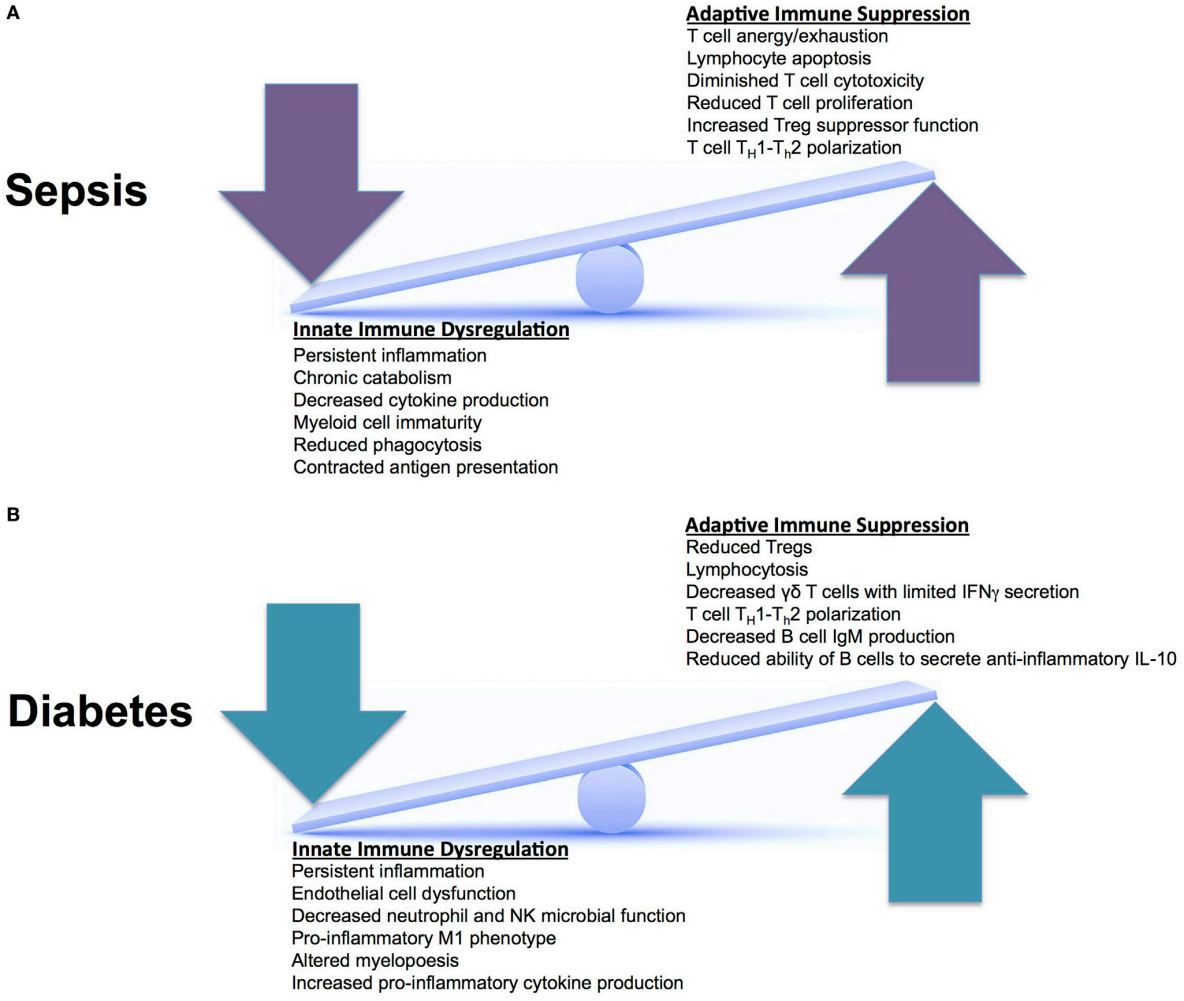
Innate versus adaptive immune responses in sepsis and Type II diabetes. **(A)** During an acute episode of sepsis, the innate and adaptive immune systems are in a constant state of fluctuation. They respond to an invading pathogen and attempt to recover homeostasis after the pathogen is cleared. This continual seesaw effect is thought to drive ongoing inflammation, facilitate organ injury, and enable infectious complications. **(B)** In diabetes, the innate and adaptive immune systems experience chronic derangements secondary to chronic inflammation, also placing these systems in constant flux. When these two systems (sepsis and diabetes) are superimposed, patients have increased morbidity and mortality; however, we do not know why. There are unclear synergistic versus antagonistic changes occurring that leads to worsened immune system perturbations and the inability to return to homeostasis.

Type II diabetes patients have an increased susceptibility to pathological infections. These patients also have some of the worst long-term morbidity and mortality. This is secondary to the inability to eradicate pathological infections. In sepsis, in addition to immune activation, a component of immune suppression concomitantly exists, which enables individuals to develop recurrent, secondary, and nosocomial infections. This leads to worse outcomes and increased long-term mortality (26). The combination of chronic immune suppression from T2D, combined with sepsis-induced immune suppression, leads to innate and adaptive immune system changes that the human body cannot overcome. As illustrated in Figure 2, both the innate and adaptive immune systems are affected in T2D and sepsis, altering homeostasis. It is not known how these aberrant pathways interact when they are superimposed. However, we do know that these superimposed pathways lead to worsened morbidity and mortality.

When looking at immune suppression in the innate immune system, there are several key pathways to mention. Neutrophils are essential for bacterial eradication. In T2D and sepsis, neutrophils display defects in chemotaxis and recruitment to sites of infection (186, 187). This leads to the reduced ability to eradicate bacteria (99). T2D-associated hyperglycemia also increases cytosolic calcium in neutrophils, which inhibits the synthesis of ATP leading to reduced chemotactic, phagocytic, and bactericidal activity. The production and release of essential effector molecules, such as ROS and cytokines, is significantly impaired leading to bacterial persistence and the development of infectious complications (133, 187, 188). In addition, T2D is associated with elevated FFAs from dysregulated carbohydrate metabolism, which cause EC dysfunction and pathological cytokine fluctuations (189). In T2D, the antioxidant systems and humoral immunity are also depressed. Furthermore, T2D predisposes patients to micro- and macrovascular comorbidities leading to environments susceptible to infections (190).

In addition to diminished innate function, adaptive immunity is similarly impaired. Splenocytes harvested from deceased sepsis patients demonstrate reduced numbers of CD4^+^ and CD8^+^ lymphocytes, due to substantial apoptosis (13). Apoptosis of lymphocytes and APCs (DCs, T cells, and B cells) is considered a hallmark of septic immune suppression (191, 192). Moreover, CD4^+^ cell loss is associated with a reduced ability to mount immune responses to viral infections after septic insults (193). However, reduced lymphocyte numbers are not just reflective of the risk for viral reactivation following sepsis. Lymphopenia 4 days after the onset of sepsis is associated with the development of secondary infections and is predictive of long-term mortality at 1 year after sepsis (194).

Several studies have examined the link between increased infectious morbidity and T2D. It is hypothesized that T2D patients are predisposed to infection due to impaired neutrophil function, decreased adaptive immune response, and dysfunctional immune cell function through high serum levels of inflammatory mediators (195). The cellular alterations observed in T2D and sepsis combine to create a chronic state of immune suppression, characterized by recurrent, secondary, and nosocomial infectious complications (196). These infectious complications often result in hospital readmissions (197–199) and poor long-term survival (200). Compared to patients without sepsis, sepsis survivors require more antibiotics, have more ICU days, and consume more hospital resources (201). T2D patients are also associated with bacterial pathogens with increased antibiotics resistance, such as MRSA, *Pseudomonas*, and *Acinetobacter*, which are associated with ICU-related mortality (202).

It is evident that sepsis induces a pathological state of immune suppression that prompts the development of secondary infections while still in the ICU setting (203). In addition, several reports demonstrate that sepsis survivors and T2D patients experience dramatically higher rates of subsequent infections long after the initial episode of sepsis has abated (204, 205). The increased hospital readmission rates due to infectious complications among T2D patients and sepsis survivors is a sign of ongoing immune suppression and dysregulation that if not corrected, diminishes life quality and durable survival. With the ever increasing, comorbidity challenged, elderly T2D population experiencing persistent inflammation, immune suppression, and immune senescence, the number of T2D sepsis survivors who develop subsequent infections is predicted to rise substantially in the next decades (200, 206).

## Immune-Modulatory Therapies in T2D

Below we will address immune modulators/modulatory pathways that deserve further consideration as disease-modifying therapeutics. These immune modulators, their proposed benefits, and some possible combinations are also listed in Table 1.

**Table 1:** Immune modulators.

Immune modulators, diabetes	IL-1 inhibition	TNF inhibition	NF-κβ inhibition	Diacerin	MCP-1 antagonism	IL-6 inhibition	Sirtuins augmentation	PPAR-γ agonists
Proposed benefit	↓ acute phase inflammation	↓ risk of developing T2	↓ release of TNF-α, IL-1B, IL-8, and MCP-1	↓ concentrations of TNF-α and IL-1B	↓ monocyte/macrophage migration/infiltration	↓ inflammation	↑ insulin secretion	↓ insulin resistance
	↓ pancreatic β-cell apoptosis		↓ hemoglobin A1c	↑ insulin secretion	↓ insulin resistance		↑ insulin sensitivity	↓ hemoglobin A1c
	↑ insulin secretion		↓ insulin clearane	↑ metabolic control				↓ macrophage concentration

**Potential cells affected**	**T cells, Lymphocytes**	**Neutrophils, macrophages, endothelial cells**	**T cells, lymphocytes**	**Neutrophils, macrophages**	**Monocytes, Macrophages**	**T cells, monocytes, neutrophils, lymphocytes**	**T cells, monocytes, neutrophils, lymphocytes**	**Macrophages**

**Immune modulators, sepsis**	**G-CSF**	**GM-CSF**	**IFNγ**	**PD-1 and PD-L1**				

Proposed benefit	↑ neutrophil and monocyte production and release	↑ neutrophil/monocyte production and function	↑ monocyte HLA-DR expression and function	↓ T cell exhaustion				
	↑ myelopoiesis and granulopoiesis	↑ monocyte/lymphocyte cytotoxicity	↓ infection and related complications	↑ lymphocyte proliferation				
		↑ T cell responses	↑ immunity against fungal infections	↑ neutrophil and monocyte cytotoxicity				
		↓ nosocomial infection acquisition		↑ opportunistic infections				
		↓ ventilator days						

**Potential cells affected**	**T cells, monocytes, neutophils, lymphocytes**	**T cells, monocytes, neutrophils, lymphocytes**	**T cells, monocytes, neutophils, lymphocytes**	**T cells, monocytes, neutrophils**				

**Proposed combinations**	**PD-1 and MCP-1**	**PD-L1 and diacerin**	**IFNγ and diacerin**					

Proposed benefit	↓ monocyte infiltration	↓ inflammation	↑ monocyte function					
	↑ lymphocyte proliferation	↑ neutrophil and monocyte cytotoxicity	↓ inflammation					
	↑ T cell function	↓ opportunistic infections	↓ fungal infections					

**Potential cells affected**	**Lymphocyte, T cells, monocytes**	**Neutrophils, monocytes**	**Monocytes**					

### IL-1

IL-1 has long been given to patients after transplantation to enhance recovery (207). Since these patients developed symptoms and signs of a systematic inflammatory reaction during treatment, subsequent research focused on blocking IL-1 during sepsis by using anakinra, a naturally occurring IL-1RA. There have been multiple controlled trials of anakinra in human sepsis. In one placebo-controlled trial, there was a reduction in 28-day all-cause mortality, but the results did not reach statistical significance (208). Attention was then turned to focus on antagonism of IL-1 during noninfectious chronic inflammatory diseases, including myeloma and rheumatoid arthritis. IL-1β antagonism is now the standard of therapy in autoinflammatory diseases (209). T2D can be classified as an autoinflammatory disease, with the innate immune system inappropriately activated due to metabolic stress leading to a chronic inflammatory disease (210). IL-1 prevents insulin secretion while promoting pancreatic β-cell death *via* apoptosis (211). In patients with T2D, there is increased expression of IL-1 expression in pancreatic β-cells with subsequent reduction in IL-1RA (212). In these patients, anakinra lowered blood glucose levels and improves β cell secretory function and insulin sensitivity, as well as reducing evidence of systemic inflammation. Just as interesting, after withdrawal of anakinra treatment, improvement in insulin secretion lasted 39 weeks (212), suggesting that the therapeutic effect IL-1 antagonism is long-lasting, perhaps due to interruption of IL-1 autoinduction (213). However, anakinra has a short half-life requiring daily administration to maintain adequate suppression of IL-1β and often causes injection-site reactions, limiting its ability to serve as a long-term therapy option (214). Subsequent studies therefore focused on humanized monoclonal antibodies, Gevokizumab, Canakinumab, and LY2189102, against IL-1β. Gevokizumab improved glycemic control (potentially by restoring insulin production) and reduced inflammation in patients with T2D (210, 215). Given the half-life of around 3 weeks, preliminary studies indicated that monthly or longer administration might be possible. Clinical trial NCT00900146 utilized Canakinumab and showed a numerical reduction in hemoglobin A1C, with a trend toward improved insulin secretion rate (216). LY2189102 improved glycated hemoglobin levels and corrected fasting and postprandial glycemia, as compared to placebo (217). In addition, just like the studies on anakinra, treatment effects were noted to be long lasting, even after treatment was stopped. These trials show the potential therapeutic benefit of inhibiting the IL-1 pathway. To further support this, a current diabetic sulfonylurea medication Glibenclamide has actually been shown as a powerful inhibitor of IL-1β in islet cells (93).

### TNF

The role of TNF in insulin resistance and T2D was first observed in 1993 (218). Numerous clinical trials have evaluated the benefits of TNF antagonism but have failed to demonstrate advantageous effects on glucose metabolism (219–221). However, these trials were underpowered, with limited patients over a short amount time, and did not account for inter-individual variations (genetic background, body weight, food intake, and exercise). Trials on TNF for other inflammatory diseases, including Crohn’s disease, rheumatoid arthritis, and psoriasis, implicate TNF blockade in altering insulin sensitivity (222, 223). Large cohort studies in patients with rheumatoid arthritis and psoriasis showed that TNF inhibition is associated with a reduction in T2D rates (224, 225). Further clinical trials specifically focusing on T2D with prolonged antagonism of TNF will likely prove to be therapeutically beneficial.

### Nuclear Factor-Kappa Beta (NF-κβ)

Lipopolysaccharides from bacterial cell walls and FFAs bind Fetuin-A to activate TLR2 and TLR4, leading to nuclear translocation of NF-κβ, which induces an inflammatory response (226, 227) through the release of TNF, IL-1β, IL-8, and MCP-1 (93). Since 2001, we have known that salsalate, a prodrug form of salicylic acid, can ameliorate T2D *via* inhibition of NF-κβ (228). Multiple trials have been completed to evaluate the potential therapeutic role of salsalate. An initial proof-of-concept study showed improvement in glycemia, decreased C-reactive protein levels, and higher adiponectin in plasma (229). Follow-up studies supported this initial observation (230, 231) with two multicenter, placebo-controlled studies, including clinical trial NCT00799643, showing that salsalate can decrease hemoglobin A1c and improve other markers of glycemic control (232, 233). However, salsalate also reduces the clearance of insulin, and thus lowers glucose concentrations through a non-inflammatory mechanism (229, 232). Metformin, a current widely accepted diabetic drug, has been shown to inhibit release of pro-inflammatory cytokines *via* IL-1β mechanisms by antagonizing NF-κβ in cells of the vascular wall as well as in macrophages (234). Metformin also inhibits the maturation of IL-1β in macrophages (235).

### Diacerein

Diacerein is a common medication for inflammatory joint disease. It decreases concentrations of cytokines such as TNF and IL-1β (236, 237). Given the benefits seen in long-term use in inflammatory joint disease, it was hypothesized that diacerein could provide benefit in T2D. The randomized, double-blind placebo-controlled clinical trial NCT01298882 showed increased insulin production and improved glycemic control after treatment with diacerein in patients who were drug naïve. Further studies investigating the mechanism of action and the role it plays in immune dysfunction could reveal a therapeutic role for diacerein in T2D patients.

### MCP-1 Antagonism

Monocyte chemoattractant protein-1 (or CCL2) is an essential chemokine active in the migration and infiltration of monocytes/macrophages (238). MCP-1 levels are increased in patients with T2D (239, 240). The gene expression of MCP-1 and its receptor CCR2 is elevated within visceral and subcutaneous adipose tissue of patients with obesity, as contrasted to lean controls (241). In addition, there is increased expression in omental fat with increased macrophage proliferation, when compared with the fat within the subcutaneous tissue (242). CCX140-B is a CCR2 antagonist. A pilot study in patients with T2D showed that administration of CCX140-B decreased placebo-corrected glycated hemoglobin (93). Multiple studies have shown that downregulation of MCP-1 cooccurs with improvement in the symptoms of T2D. These results implicate a close relationship and support further studies that investigate the role of MCP-1 as a therapeutic target (240).

### IL-6

IL-6 is a one of the main cytokines that is responsible for an inflammatory processes and responses. It is produced by macrophages, T cells, osteoblasts, kidney cells, muscle cells, and adipocytes (243). It has pleiotropic effect on glucose metabolism that is dependent on tissue type and the surrounding milieu. Increased levels of IL-6 are associated with obesity, T2D, and cardiovascular disease (244). Under specific conditions, IL-6 may either decrease or enhance insulin resistance, as well as improve glucagon-like peptide-1-mediated insulin section. In the paradigm of inflammation within obesity, it is hypothesized that IL-6 enhances the prevailing inflammation, thus precipitating insulin resistance and leading to further micro- and macrovascular complications (245).

### Sirtuins

Sirtuins represent a class of NAD^+^-dependent deacetylases that have a wide array of biological functions, one being to coordinate the body’s reaction to caloric intake. Sirtuins are associated with metabolic disorders (246) and play a critical role in restoring homeostasis during stress responses (247). Emerging evidence supports that failure to maintain homeostasis during metabolism and bioenergy reprogramming result in acute and chronic inflammatory disease (247). In obesity, there is a decrease in sirtuin 1 levels and activity. This is likely secondary to upregulation of peroxisome proliferator-activated receptor gamma (PPAR-γ) genes that regulate fatty acid uptake and triglyceride synthesis in mature adipocytes (248). Increased sirtuin 1 expression and activation is associated with increased insulin secretion (249). There are substantial data to support that increased sirtuin 1 activity counters obesity, the metabolic syndrome, and T2D with or without obesity (247) making it a desirable therapeutic target.

### Peroxisome Proliferator-Activated Receptor Gamma

A current antidiabetic therapeutic group, the thiazolidinediones which include rosiglitazone and pioglitazone, are PPAR-γ agonists. PPAR-γ is a type II nuclear receptor found mainly in macrophages, adipose tissue, and in the colon. These drugs effectively improve insulin resistance and reduce hemoglobin A1c though multiple mechanisms. One mechanism is that they can inhibit pro-inflammatory pathways leading to decreased macrophage concentration in adipose tissue (250, 251). The overall clinical effect from the improved insulin resistance and anti-inflammatory effects of these agents are not clearly defined; however, they reveal multiple mechanistic pathways to further evaluate (252).

## Immune-Modulatory Therapies in Sepsis

### Granulocyte Colony-Stimulating Factor (G-CSF) and GM-CSF

Granulocyte colony-stimulating factor stimulates the production of stem cells, progenitors, and granulocytes (253). Two randomized controlled human trials with recombinant G-CSF were performed to test its effect on neutrophil production, maturity, and overall function. Although an increase in blood leukocyte counts was observed, there was no improvement in 28-day patient mortality (254, 255). This makes one wonder if a longer study therapy or observation time would have changed the investigation outcomes. Given the ongoing and continuous alterations observed in granulocyte production, myelopoiesis, and neutrophil function in T2D and septic patients, prolonged G-CSF administration may be efficacious for improved immune surveillance, infection eradication, tissue regeneration, and survival during sepsis.

GM-CSF is an additional cytokine that enhances stem cells to differentiate into macrophages, monocytes, and neutrophils (256). In one study, ventilator-dependent septic patients who were prescribed GM-CSF during the immune suppressive phase had fewer days on the ventilator and within the ICU (257, 258). Recombinant GM-CSF treatment in septic children improved lymphocyte TNF production and significantly reduced hospital-associated infections (259). Further evidence for GM-CSF therapy from a meta-analysis of over 12 clinical studies using GM-CSF or G-CSF showed that treatment with either reduces infectious complications (260). In light of the fact that 70–80% patients who succumb to sepsis harbor persistent, chronic, ongoing, or secondary infections (13), G-CSF or GM-CSF combined with other immune regulators may bolster immune response and eradicate infection in septic T2D populations, potentially improving overall survival (254, 261).

### Interferon Gamma

Interferon gamma is the sole protein within the family of type II interferons. Adequate IFNγ production and signaling is critical for appropriate immune targeting of microbial invaders. IFNγ is also a central inducer of macrophage activation, stimulating class I MHC expression (141). Patients with severe sepsis treated with recombinant IFNγ demonstrate reversal of sepsis-induced monocytic dysfunction, as well as having better overall survival (262). It is important to note that even though the patient population of most trials involving IFNγ were mixed cohorts of severe trauma patients, the largest study reports a clear decrease in mortality due to infections (263). A recent report on severe trauma patients shows that 42 of 63 genes were within the interferon pathway and differentially expressed in patients with uncomplicated versus complicated outcomes. Recombinant IFNγ treatment was also able to partially restore immune metabolic defects associated with immune paralysis in humans after sepsis, further suggesting that IFNγ therapy after sepsis may benefit a multitude of cellular immune functions (264). IFNγ is a very promising agent if it is targeted to specific patient populations, such as T2D patients who have immune suppression, adaptive immune dysfunction, and chronic inflammation.

### Programmed Cell Death Protein-1 and Ligand (PD-1 and PD-L1)

The PD-1 protein is expressed on myeloid lineage cells and most B- and T-lymphocytes, while its ligand (PD-L1) is expressed universally on monocytes, macrophages, epithelial cells, ECs, and DCs (265). Its ultimate effect is inhibitory, reducing CD8^+^ T cells from proliferating or accumulating in lymphoid organs. PD-1 becomes upregulated during viral infections and cancer states and is associated with “T cell exhaustion from prolonged periods of exposure to self-antigens” (266). Subsequently, patients in septic shock exhibit higher levels of PD-1 and PD-L1 on their monocytes and T-lymphocytes (267). Anti-PD-1 and anti-PD-L1 have demonstrated encouraging results in clinical trials on human with viral infection or cancer (267). Studies have demonstrated that upregulation of granulocyte PD-L1 potentiates lymphocyte apoptosis *via* contact inhibition, which correlates with outcome (268). Given PD-1 and PD-1L’s positive effect on adaptive immunity as well as tumor growth, they both could be used as biomarkers of immune suppression from sepsis. They are also potential targets to ameliorate adaptive immune dysfunction or increase overall survival in the long-term (9).

## Conclusion

Type II diabetes is a disease of altered immunity that results in protracted inflammation, immune suppression, and significant infection morbidity. Clinically, it is obvious that patients with T2D are more susceptible to infections. In sepsis, despite the best goal-directed therapies that control hyperglycemia, administer antibiotics early, and prevent organ damage, T2D patients still have worse morbidity and mortality for reasons that are poorly understood. However, the link between the two appears to be the dysregulated immune pathways. We believe that immune-modulatory therapies that are strategically introduced and influence the interdigitating immune derangements between these two diseases have the potential to substantially improve the overall morbidity and mortality that these individuals experience.

## Author Contributions

All authors have made substantial contributions to all phases of manuscript development. MD and LF conceived the larger project. FF, KH, and PW helped conceive the focus of the paper. LF drafted the first version, figures, and table, with all authors providing substantive and editorial feedback on multiple revisions. We have all approved the final version prior to submission.

## Conflict of Interest Statement

The authors declare that the research was conducted in the absence of any commercial or financial relationships that could be construed as a potential conflict of interest.
